# In-silico assessment of phytochemical derivatives generated using CHEESE webserver for advancement of druggable candidate in pancreatic cancer therapy

**DOI:** 10.1007/s40203-025-00536-w

**Published:** 2026-01-14

**Authors:** Christopher Busayo Olowosoke, Felix Oluwasegun Ishabiyi, Amal Bouribab, Aqsa Munir, Blessing Awoyemi, Winifred Njideka Nsofo, Amorha Chizoba Christabel, Jonah Ojochogwu Joy, Samir Chtita, Victor Omoboyede, Prosper Obed Chukwuemeka

**Affiliations:** 1 School of Science, RMIT University, PO Box 71, Melbourne, Bundoora, 3083 Australia; 2 Department of Biotechnology, School of Life Sciences (SLS), Federal University of Technology Akure, P.M.B 704, Akure, Ondo State Nigeria; 3 Research Development Unit, Institute of Bioinformatics and Molecular Therapeutics, Osogbo, Osun State Nigeria; 4 Faculty of Pharmacy, University of Ibadan, Ibadan, Oyo State Nigeria; 5 Laboratory of Analytical and Molecular Chemistry, Faculty of Sciences Ben M’Sik, Hassan II University of Casablanca, Casablanca, Morocco; 6 Multan, Pakistan; 7 Naples, Italy; 8 Department of Microbiology, School of Life Sciences (SLS), Federal University of Technology Akure, P.M.B 704, Akure, Ondo State Nigeria; 9 Department of Biochemistry, School of Biological Sciences, Federal University of Technology Owerri, Owerri, Imo state Nigeria; 10 Department of Medical Biochemistry, Faculty of Basic Medical Sciences, University of Abuja, Federal Capital Territory, Nigeria; 11 Department of Microbiology, Faculty of Natural Sciences Kogi State University Anyigba, Anyigba, Kogi State Nigeria; 12 Department of Biochemistry, School of Life Sciences (SLS), Federal University of Technology Akure, P.M.B 704, Akure, Ondo State Nigeria

**Keywords:** CHEESE webserver, Phytochemicals, EZH2, ADMET, Molecular docking, Molecular dynamics simulation

## Abstract

Research efforts for pancreatic cancer (PC) therapy has led to investigations of numerous therapeutic targets, yet there are still limited efficacy outcomes. In 2021, the annual PC cases of 508,533 resulted in mortality of 505,752 for both sexes according to GLOBOCAN. In order to address this burden, enhancer of zeste homolog 2 (EZH2); an epigenetic regulator implicated in various cancers, has been an attractive target, due to promising tumor-suppressive effects in both preclinical and clinical studies. This effect was observed from drugs that have strong affinity towards EZH2, but there is need to improve the structural moieties for better interaction towards this protein. In this study, we employed a structure-based drug discovery approach using CHEESE webserver for rapid ligand-based screening to identify and evaluate phytochemical derivatives for their potential to bind EZH2. Five phytochemicals, namely Moracin P, Naringenin 5-rhamnoside, Pinostrobin 5- glucoside, Phytocassane A, and Sakuranin with best performance against EZH2-PPARs from our previous study was used to generate top ten new derivatives each. The derivatives were subjected to molecular docking, pharmacokinetic, and toxicity predictions. The top-performing derivative interacting with EZH2 were further subjected to ADMET profiling with favorable pharmacokinetic, toxicity properties, and meeting key drug-likeness criteria. Molecular docking results revealed that several derivatives of Moracin P, Naringenin 5-rhamnoside, and Phytocassane A displayed higher predicted binding affinities (− 6.4 to − 8.2 Kcal/mol) compared to the parent template previously assessed for EZH2, engaging critical residues through hydrogen bonds and hydrophobic interactions. Molecular dynamic simulation (MDS) over 200 ns further confirmed the stability of selected protein-ligand complexes, with Moracin_P7 and Pinostrobin 5-glucoside_5 exhibiting high degrees flexibility within the first 100 ns, but remained stable for the last 100 ns, while contact analysis highlighted consistent interactions with the active site residues Gln653, Asp657, Asp664, Ser669, Asn673, Phe678, His711 and Tyr731. Conclusively, these findings provide evidence that CHEESE webserver is suitable to generate phytochemical derivatives that can be explored as feasible candidate in EZH2 inhibitory study and laying the groundwork for further in-vitro and in-vivo validation.

**Supplementary Information:**

The online version contains supplementary material available at 10.1007/s40203-025-00536-w.

## Introduction

Pancreatic cancer (PC) is a lethal malignancy of the pancreas and arises from genetic alterations in oncogenes and tumor suppressor genes in the pancreatic cells, which initiate aberrant cellular signaling, and ultimately uncontrolled proliferation and tumorigenesis (Amaral et al. 2023). PC remains one of the leading causes of global cancer-related deaths, particularly in developed countries (Sung et al. 2021). According to the Global Burden of Disease Study 2021, PC remains one of the most lethal malignancies worldwide, with an estimated 508,533 new cases and 505,752 deaths reported in 2021 alone. This reflects a substantial increase from 1990, when 207,905 new cases and 211,613 deaths were recorded (Li et al. 2025). This rising global burden has been attributed to several factors, including an aging population, environmental exposures, occupational hazards, and metabolic disorders such as diabetes and obesity, which are frequently linked to dietary and lifestyle choices (Boonhat et al. 2023; Ruze et al. 2023). Additionally, genetic predisposition also plays a role, with familial clustering increasing individual risk (Klatte et al. 2022). Notably, over 80% of PC cases occur in individuals over 60, while it remains rare among those under 45 (Rawla 2019). Despite scientific and technological advancements that have improved the rate of early detection, projections estimate that PC incidence may rise to 355,317 new cases by 2040 due to global population growth (Rahib et al. 2021). Consequently, efforts to further unravel mechanisms that drive the development, progression, and therapy resistance of these lethal diseases remain ongoing.

Recent advances in molecular oncology have underscored the importance of epigenetic regulators in the pathogenesis of PC. Among these, the Enhancer of Zeste Homolog 2 (EZH2) and its homolog EZH1, which are key components of the Polycomb Repressive Complex 2 (PRC2), have been identified as crucial drivers of PC progression. While these enzymes are involved in gene silencing during cell differentiation and development, their dysregulation in cancer cells contributes to tumorigenesis (Parreno et al. 2022). EZH2 has been shown to regulate multiple key signaling pathways, including NF-κB, Wnt, NOTCH, and RAS, consequently influencing several biological processes critical to oncogenesis, such as proliferation, apoptosis, migration, and metastasis (Li et al. 2023; Yu et al. 2023). Among the aforementioned pathways, the Wnt signaling pathway is particularly significant in PC, as it plays a central role in driving epithelial-mesenchymal transition and contributing to chemoresistance (Patel et al. 2019; Ram Makena et al. 2019). The progression of PC is further fueled by the activation of the Wnt pathway through non-canonical ligands, such as CDK8, GATA6, R-spondin, K-ras, MUC1, R-spondin2, and MCU4, among others, consequently exacerbating tumorigenic processes and facilitating cancer cell survival and metastasis (Wang et al. 2009).

Interestingly, inhibition of EZH2 has shown promising tumor-suppressive effects in both preclinical and clinical studies. Exemplifying this is a study by Hu et al. in which they demonstrated that Bezafibrate, a pan-peroxisome proliferator-activated receptor (pan-PPAR) agonist commonly used in diabetes management, enhances the efficacy of GSK126, a selective EZH2 inhibitor, in pancreatic cancer cells (Hu et al. 2021). The combination of GSK126 and Bezafibrate significantly promoted apoptosis and inhibited cell proliferation. This synergistic effect was linked to the suppression of Wnt/β-catenin signaling, suggesting that the EZH2–PPAR axis could serve as a potential therapeutic target in pancreatic cancer (Hu et al. 2021). Building on this foundation, Olowosoke et al. identified five phytochemicals widely known for their diverse pharmacological properties as promising EZH2 inhibitors. These phytochemicals include Moracin P, Naringenin 5-rhamnoside, Phytocassane A, Pinostrobin 5-glucoside, and Sakuranin, and they exhibited stronger predicted binding affinities for EZH2 than GSK126 and bezafibrate (Olowosoke et al. 2023). Despite several efforts, only Tazemetostat has been approved for clinical usage as EZH2 inhibitor, with several such as CPI-1205, GSK126, and valemetostat still in clinical trials, and their translation to solid tumors like pancreatic cancer has been limited by modest efficacy, emergence of resistance mechanisms, lack of robust predictive biomarkers, and potential toxicity from off-target epigenetic effects (Yang et al. 2023). Although the templates from our previous study for EZH2 is promising, using traditional methods to design new derivatives is slow, expensive and can be impractical for routine applications. Therefore, we sought the best phytochemical template and built new structural moieties to improve on the outcome of affinity and interaction based on similarity and for rapid ligand-based screening from a newly commission webserver known as CHEESE. By using CHEESE, we can revolutionize drug discovery, reduce candidate compounds failure and promote scalable chemicals that can be easily synthesized by medicinal chemists, or available and accessible from chemical vendors due to its leverages and interacts with several major chemical databases and technologies. This will aid in expansion of the compound candidate library that can be tested in addressing the gap in effective EZH2-targeting therapies.

## Materials and methods

### Materials

In this study, some of the software and databases employed are as follows: database— CHEESE, PubChem, ENAMINE-REAL, ZINC 15, and Protein Data Bank. Software— Chimera, Discovery Studio (DS) version 21.1, Ligplot v2.2.4, PyMol 1.3, PyRx. The exhaustive computer-aided procedures from the webservers and software were carried out on a Dell Latitude E7270, x64-based PC, Intel (R) Core (TM) i7–6600U2.60 GHz, 8GB RAM system.

### Methods

#### The substructure of phytochemical template retrieval

The SMILES of the five phytochemicals identified from a previous computational study by Olowosoke et al. (Olowosoke et al. 2023) as having higher inhibitory potentials than GSK-126 for EZH2 were retrieved from the PubChem database (https://pubchem.ncbi.nlm.nih.gov/) (Kim et al. 2023) and were utilized to identify a new set of substructural analogues based on the parent templates, leveraging the CHEESE webserver (https://cheese.deepmedchem.com/) (Bolcato et al. 2022; Kumar and Zhang 2018). The generated analogues substructure ID was based on “very accurate” search type, and the top ten structure sdf. were retrieve from ENAMINE-REAL (https://enaminestore.com/) and ZINC 15 (https://zinc15.docking.org/substances/home/) databases (Supplementary file 1–5).

#### Protein retrieval and preparation

According to Antonysamy et al. the overexpression and mutation of EZH2 are associated with the incidence and aggressiveness of various cancers, and the appropriate x-ray crystallographic structure with structural context for clinically significant mutation found on the target protein, EZH2 was (PDB: 4MI5) (Antonysamy et al. 2013). This was downloaded from the Protein Data Bank (PDB) (https://www.rcsb.org/) in PDB format (Ahmad et al. 2025; Berman 2000; Finogenova et al. 2020). The retrieved structure was subjected to a rigorous preparatory procedure using the Discovery Studio and Chimera v1.16 Software as reported by Olowosoke et al. (Olowosoke et al. 2023).

#### Molecular docking simulation

A site-specific docking protocol was employed to predict the binding affinity and assess the interaction of the identified derivatives with the active site of EZH2. The active site of EZH2 was first outlined using Discovery Studio, followed by creating a grid box to define the binding region. The grid box was set with dimensions of x = 26.65 Å, y = 16.52 Å, and z = 18.29 Å, with the center coordinates at x = 255.81 Å, y = 276.25 Å, and z = 250.98 Å. A single run of docking simulations were carried out using AutoDock Vina, integrated within the PyRx software, with an exhaustiveness value of 8 to ensure thorough sampling of the binding site (Olukunle et al. 2023). Following the completion of the docking simulations, the interactions between the phytochemical derivatives and the protein target with RMSD of 0.000 Å were visualized using Ligplot v2.2.4 for 2D interaction mapping and PyMOL for 3D visualization of the binding poses.

#### Absorption, distribution, metabolism, extraction, and toxicity (ADMET) profiling

To assess the viability of the identified derivatives for therapeutic purposes, the ADMET profiling technique was employed to conduct a thorough screening, evaluation, and prediction of the ADMET properties of the compounds. These properties were assessed by utilizing the CHEESE modeller incorporated into the CHEESE webserver. This platform examines the bioavailability, aqueous solubility, pharmacokinetics, lipophilicity, and drug-likeness of compounds, and we used only the best docked from the derivatives of Moracin P, Naringenin 5-rhamnoside, Phytocassane A, Pinostrobin 5-glucoside, and Sakuranin. ADMET analysis via computational methods plays a significant role in drug discovery as it aids in identifying compounds to prioritize for in vitro and in vivo studies to avoid failure in the latter phase of drug discovery due to toxicity issues (Omoboyede et al. 2023).

#### Molecular dynamics simulation

Molecular dynamics simulation (MDS) were performed using Desmond, a high-performance MD engine integrated within the Schrödinger suite, to investigate the binding stability of ligands and the conformational dynamics of the EZH2 protein-ligand complexes (Rossafi et al. 2025). The simulations were conducted via the Schrödinger Maestro interface over a 200 ns, as previously described (Parida et al. 2020; Sheikh et al. 2023). Prior to simulation, the receptor structures were prepared by removing overlapping water molecules and solvating the systems with TIP3P crystallographic water molecules, using orthorhombic periodic boundary conditions (Bouribab et al. 2025). A 10 Å buffer region was defined for all systems, and neutralization was achieved by adding Na⁺ or Cl⁻ counterions to maintain a physiological salt concentration of 0.15 M (Rossafi et al. 2025). The systems were first subjected to 1 ns of NVT equilibration, stabilizing the temperature under a constant volume (Guerguer et al. 2025). This was followed by NPT equilibration at 300 K and 1 bar pressure, with the production MD run lasting 200 ns. These equilibration and production steps, governed by accurate thermostat and barostat algorithms, ensured a stable and reliable simulation environment for assessing protein-ligand interactions.

## Results

### Physicochemical properties assessment of phytochemical derivatives

The physicochemical properties of the identified structural analogues are presented in Table 1. As evident in Table 1, the physicochemical profiling of the derivatives revealed varied degrees of drug-likeness across the compound classes, based on key parameters including bioavailability (BA), topological polar surface area (TPSA), lipophilicity (ClogP), solubility, and molecular flexibility (rotatable bonds).

Moracin P derivatives exhibited generally favorable physicochemical profiles, with bioavailability scores ranging from 0.984 to 0.999, indicative of good oral absorption potential. Most compounds had moderate TPSA values (75.96–113.4 Å^2^), acceptable for passive permeability, and rotatable bond counts within the ideal range (2–7). However, lipophilicity (ClogP) exceeded the optimal threshold in many derivatives. Solubility was also poor for most derivatives (≤ − 4.3), except for compound 3 (− 4.87), which presented a slightly improved solubility profile (Table 1). Conversely, among the Naringenin 5-rhamnoside derivatives, compounds displayed high TPSA values, possibly due to the presence of sugar moieties, which may reduce passive permeability. All derivatives showed moderate-to-high bioavailability (0.579–0.858) and good molecular weight ranges (< 400 Da). However, lipophilicity was generally poor (CLogP < 0.5 in most derivatives), suggesting possible issues with membrane permeability. Solubility values were near the lower threshold of acceptability (e.g., − 5.10 to − 2.81), reflecting moderate water solubility (Table 1). As opposed to Moracin P and Naringenin 5-rhamnoside derivatives, Phytocassane A derivatives demonstrated more consistent properties across derivatives. Notably, they all showed low-to-moderate TPSA (57.53–74.6 Å^2^), minimal rotatable bonds, and high lipophilicity in several cases (CLogP > 3.0). Bioavailability was lower compared to other classes (0.07–0.517), suggesting possible limitations in oral exposure. However, good solubility (− 3.48 to − 4.22) and acceptable molar refractivity (92–98 Å^2^) support their potential as leads for further optimization. Pinostrobin 5-glucoside derivatives showed variable performance. Several derivatives exceeded the acceptable lipophilicity range, while others, like derivative 6, had more balanced profiles (BA = 0.987, CLogP = 1.81). TPSA values remained within a drug-like range (64.6–117.2 Å^2^), and solubility was moderately poor (e.g., compound 5: − 5.53), but acceptable in others. Most compounds had appropriate molecular weights (< 450 Da) and rotatable bond counts, indicating conformational flexibility compatible with drug-like behaviour. Sakuranin derivatives exhibited promising properties, especially derivatives 6–10, which showed high bioavailability (≥ 0.963), moderate TPSA (~ 91.3 Å^2^), acceptable molecular weight (396.1 Da), and good CLogP values (3.70–3.71). While these compounds had slightly poor solubility (− 5.35 to − 5.43), they fell within tolerable limits, and their overall profile suggests favorable membrane permeability and oral absorption. In contrast, derivatives 1–5, despite possessing ideal TPSA and donor/acceptor counts, showed poor lipophilicity (CLogP = 0.286) and were predicted to have suboptimal bioavailability (0.304).

**Table 1:** Phytochemical derivatives and their physicochemical properties based on CHEESE webserver

Phytochemicals	Derivative number	Sub-structure ID	BA	Hydrogen acceptors	Hydrogen donors	Rotatable bonds	TPSA	Molecular weight	Clog *P*	Solubility	Lipophilicity	Molar refractivity
Moracin P	1	Z1642390909	1	6	0	3	81.8	328.05178	3.61764	− 6.706	2.916	87.4954
	2	Z31323896	1	6	0	3	94.94	312.07462	3.14914	− 5.444	2.877	81.8844
	3	Z1123898030	0.984	5	1	3	75.96	339.05653	3.2795	− 4.868	0.788	90.3513
	4	Z729909024	0.999	8	0	7	100.26	388.07291	3.7982	− 6.423	3.414	100.3594
	5	Z729924972	0.997	8	0	3	100.26	421.92082	3.492	− 5.64	2.993	91.8444
	6	Z1642391329	0.985	8	0	4	108.1	358.02596	2.9261	− 6.201	2.944	89.3304
	7	Z31309984	0.999	6	0	3	94.94	298.05897	2.84072	− 5.236	2.865	77.1474
	8	Z4562267692	0.998	5	0	4	38.25	327.14053	3.8094	− 4.426	2.443	94.859
	9	Z3996032430	0.999	6	0	2	77.83	300.11101	3.21802	− 4.344	3.234	79.254
	10	Z31317504	0.997	8	0	7	113.4	372.09575	3.3297	− 5.817	3.121	94.7484
Naringenin 5− rhamnoside	1	ZINC001226496196_a	0.579	7	2	4	102.29	396.1209	3.7925	− 5.108	1.577	102.0291
	2	ZINC001226496195_a	0.579	7	2	4	102.29	396.1209	3.7925	− 5.108	1.577	102.0291
	3	ZINC001226496196	0.734	7	1	4	105.12	395.1136	3.1605	− 4.41	0.41	99.8103
	4	ZINC001226496195	0.734	7	1	4	105.12	395.1136	3.1605	− 4.41	0.41	99.8103
	5	ZINC001217990789	0.858	5	3	3	92.6	384.1806	2.545	− 2.807	− 0.182	103.0035
	6	ZINC001217990790	0.858	5	3	3	92.6	384.1806	2.545	− 2.807	− 0.182	103.0035
	7	ZINC001217990791	0.858	5	3	3	92.6	384.1806	2.545	− 2.807	− 0.182	103.0035
	8	ZINC001217990788	0.858	5	3	3	92.6	384.1806	2.545	− 2.807	− 0.182	103.0035
	9	ZINC001226495729	0.706	7	2	4	102.29	396.1209	3.7925	− 5.16	2.878	102.0291
	10	ZINC001226495730	0.706	7	2	4	102.29	396.1209	3.7925	− 5.16	2.878	102.0291
Phytocassane A	1	ZINC000263609967	0.265	4	2	1	74.6	348.2301	2.8891	− 3.69	2.373	93.7546
	2	ZINC000263609968	0.265	4	2	1	74.6	348.2301	2.8891	− 3.69	2.373	93.7546
	3	ZINC000263609966	0.265	4	2	1	74.6	348.2301	2.8891	− 3.69	2.373	93.7546
	4	ZINC000263609969	0.265	4	2	1	74.6	348.2301	2.8891	− 3.69	2.373	93.7546
	5	ZINC000253497334	0.517	3	2	1	57.53	346.2508	3.8762	− 4.217	3.4	97.8876
	6	ZINC000253497332	0.517	3	2	1	57.53	346.2508	3.8762	− 4.217	3.4	97.8876
	7	ZINC000253497333	0.517	3	2	1	57.53	346.2508	3.8762	− 4.217	3.4	97.8876
	8	ZINC000004612836	0.517	3	2	1	57.53	346.2508	3.8762	− 4.217	3.4	97.8876
	9	ZINC000257346268	0.07	4	2	1	74.6	346.2144	2.8092	− 3.481	2.568	93.7306
	10	ZINC000257346270	0.07	4	2	1	74.6	346.2144	2.8092	− 3.481	2.568	93.7306
Pinostrobin 5− glucoside	1	PV-004802326878	0.999	5	1	6	80.76	395.1845	3.5948	− 4.391	2.74	106.5547
	2	Z3797662750	0.998	7	1	6	98.58	385.175	1.9382	− 4.05	1.178	98.7037
	3	Z2160012302	0.999	7	0	5	83.41	355.1532	4.42012	− 4.931	3.756	94.021
	4	PV-007119978357	1	4	1	5	64.63	387.1282	3.4575	− 5.13	2.797	96.6472
	5	Z2044967864	0.998	9	1	7	117.19	426.2016	3.29864	− 5.526	3.02	111.3442
	6	PV-006581043059	0.987	5	2	6	88.1	348.1685	1.8178	− 2.822	1.055	90.4285
	7	Z3499097591	0.987	5	2	6	88.1	348.1685	1.8178	− 2.822	1.055	90.4285
	8	Z2925439329	0.987	5	2	6	88.1	348.1685	1.8178	− 2.822	1.055	90.4285
	9	Z1869550624	0.987	5	2	6	88.1	348.1685	1.8178	− 2.822	1.055	90.4285
	10	PV-007821800323	1	6	2	5	80.68	388.1457	3.575	− 5.078	3.348	103.977
Sakuranin	1	ZINC000005742781	0.304	10	5	5	155.14	448.137	0.286	− 2.731	1.45	107.8125
	2	ZINC000005742784	0.304	10	5	5	155.14	448.137	0.286	− 2.731	1.45	107.8125
	3	ZINC000005742783	0.304	10	5	5	155.14	448.137	0.286	− 2.731	1.45	107.8125
	4	ZINC000005742785	0.304	10	5	5	155.14	448.137	0.286	− 2.731	1.45	107.8125
	5	ZINC000016951757	0.304	10	5	5	155.14	448.137	0.286	− 2.731	1.45	107.8125
	6	ZINC001229482505	0.963	7	1	4	91.29	396.1209	3.7054	− 5.427	1.832	102.2993
	7	ZINC001229482503	0.963	7	1	4	91.29	396.1209	3.7054	− 5.427	1.832	102.2993
	8	ZINC001229482501	0.963	7	1	4	91.29	396.1209	3.7054	− 5.427	1.832	102.2993
	9	ZINC001229482499	0.963	7	1	4	91.29	396.1209	3.7054	− 5.427	1.832	102.2993
	10	ZINC001229476302	0.97	7	1	4	91.29	396.1209	3.7054	− 5.355	1.631	102.2993

Bioavailability; (> 0.7: good, 0.3–0.7: average, 0.0–0.3: poor), Solubility; (4.0–0.5: good, < − 4.0 or > 0.5: poor), Lipophilicity; (0.0–3.0: good, < 0.0 or > 3.0: poor). TPSA: Topological surface area, BA: bioavailability,

### Structural derivatives exhibited higher predicted binding potential against EZH2

Analysis of the top-ranked docking poses with RMSD 0.000 Å revealed that some derivatives of the parent phytochemicals exhibited improved binding affinities toward EZH2 (Table 2). Among the Naringenin 5-rhamnoside derivatives, Naringenin 5-rhamnoside_1–4 recorded the highest predicted binding affinity with a docking score of − 8.2 Kcal/mol, surpassing both their parent and other analogues. Similarly, the derivative Pinostrobin 5-glucoside_5 demonstrated a higher predicted binding affinity with a docking score of − 8.1 Kcal/mol, relative to other analogues with docking scores less than − 7.7 Kcal/mol. Moracin P derivatives also showed consistency, with P2 and P7 exhibiting docking scores of − 7.7 Kcal/mol, slightly outperforming the original compound, which had a docking score of 7.6 Kcal/mol, whereas others like P9 and P5 exhibited weaker predicted binding affinities. In contrast, the predicted binding affinities of Phytocassane A and Sakuranin derivatives showed minimal variation across analogues, clustering around − 7.1 to − 7.2 Kcal/mol.

**Table 2:** The predicted binding affinities of the parent phytochemicals and their structural derivatives docked against EZH2 at RMSD 0.000 Å

Phytochemical derivatives	Binding affinity (Kcal/mol)
*Moracin* *P*	− 7.6
Moracin P 1	− 7.5
Moracin P 2	− 7.7
Moracin P 3	− 7.0
Moracin P 4	− 7.0
Moracin P 5	− 6.9
Moracin P 6	− 7.6
Moracin P 7	− 7.7
Moracin P 8	− 7.2
Moracin P 9	− 6.8
Moracin P 10	− 7.2
*Naringenin 5− rhamnoside*	− 8.0
Naringenin 5-rhamnoside (1–4)	− 8.2
Naringenin 5-rhamnoside (5–8)	− 7.8
Naringenin 5-rhamnoside (9 & 10)	− 7.8
*Phytocassane A*	− 6.8
Phytocassane A (1–4)	− 7.2
Phytocassane A (5–8)	− 7.2
Phytocassane A (9 & 10)	− 7.1
*Pinostrobin 5− glucoside*	− 7.7
Pinostrobin 5− glucoside 1	− 7.6
Pinostrobin 5− glucoside 2	− 7.1
Pinostrobin 5-glucoside 3	− 7.3
Pinostrobin 5-glucoside 4	− 7.5
Pinostrobin 5-glucoside 5	− 8.1
Pinostrobin 5-glucoside (6–10)	− 6.4
*Sakuranin*	− 7.6
Sakuranin (1–5)	− 7.2
Sakuranin (6–9)	− 7.2
Sakuranin 10	− 7.7

### Interaction profiling

**Fig. 1:**
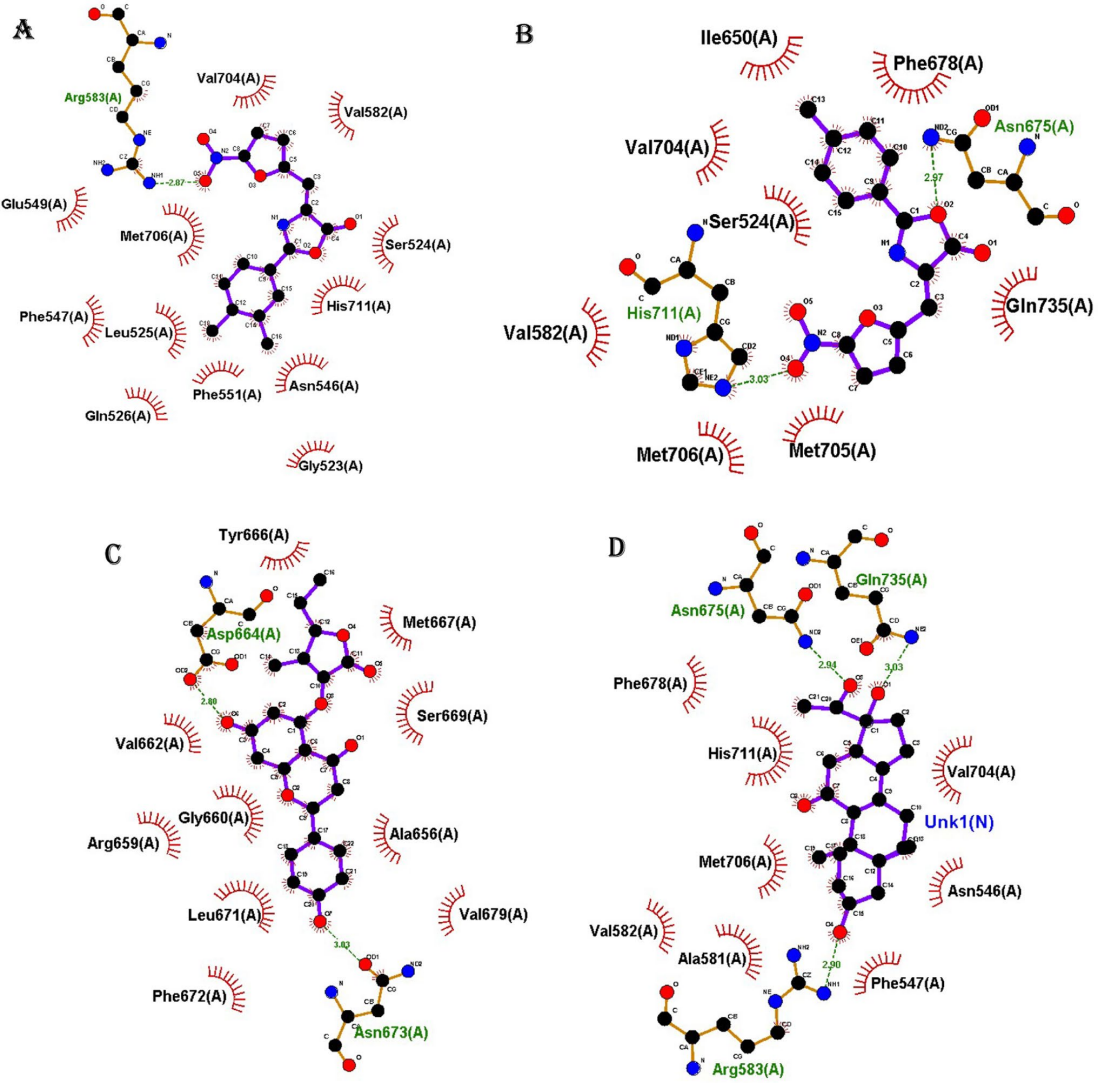

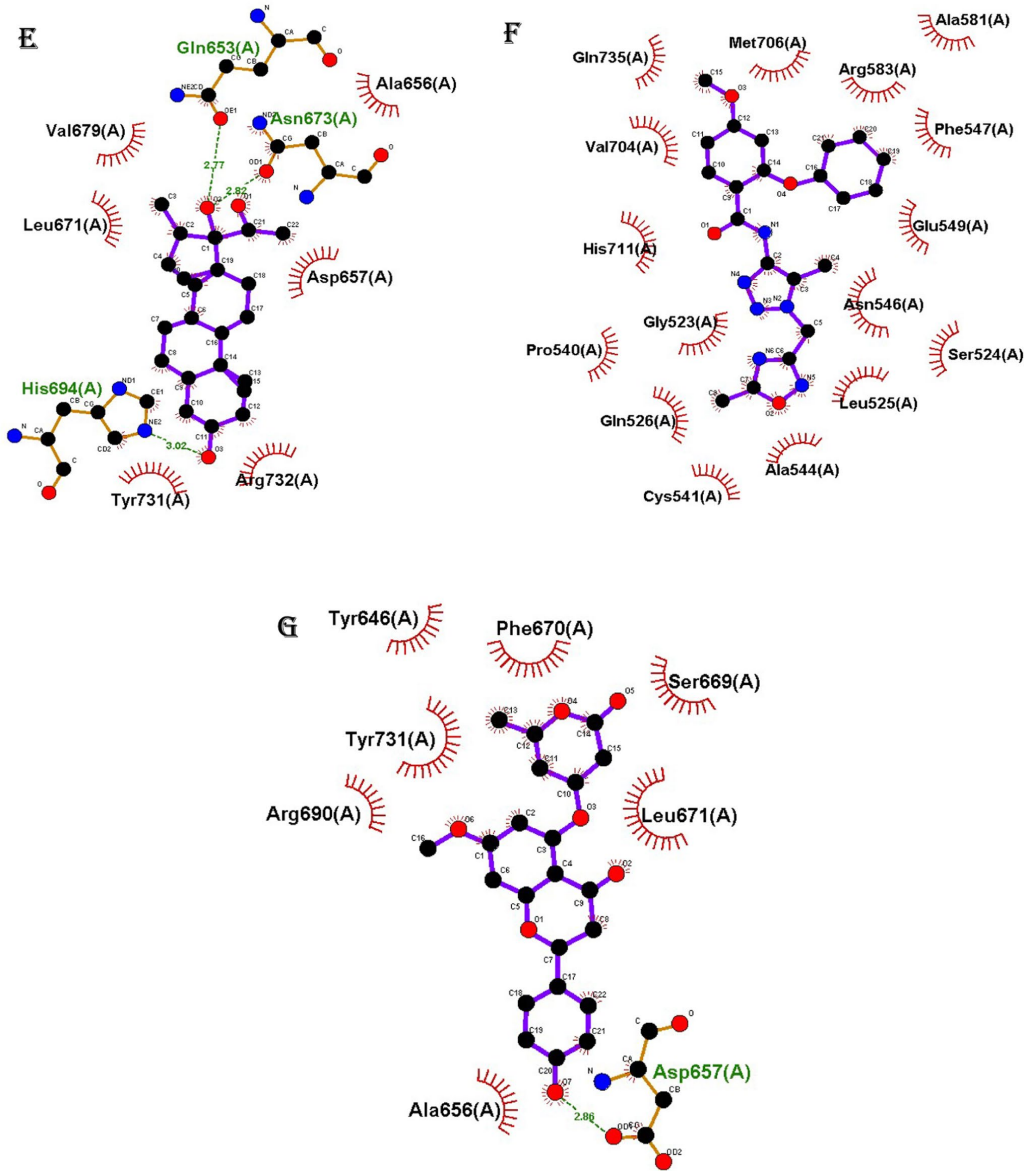
Molecular interaction profiles of selected compound derivatives with the EZH2 active site. **A**, **B** Moracin P2 and Moracin P7; **C** Naringenin 5-rhamnoside_1; **D–E** Phytocassane A_1 and Phytocassane A_5; **F** Sakuranin_6. Each compound exhibited distinct binding interactions, including hydrogen bonds (green dashed lines) and hydrophobic contacts (visualized as surrounding residues), with key amino acids in the EZH2 binding pocket

The interaction patterns formed by different scaffolds of selected phytochemical derivatives with amino acid residues within the EZH2 active site were profiled for only the best and top-ranked, and the results are presented in Fig. 1. Several derivatives demonstrated strong binding potential through key molecular interactions. Notably, selected Moracin P (Fig. 1A, B) and Naringenin (Fig. 1C) derivatives established both hydrogen bonds and extensive hydrophobic contacts with critical residues. Phytocassane A derivatives similarly engaged multiple hydrogen bonds alongside hydrophobic interactions as presented in Fig. 1D, E. In contrast, the selected Sakuranin derivative formed fewer hydrogen bonds but maintained several hydrophobic contacts, suggesting a distinct yet potentially effective binding mode (Fig. 1F).

### **In-silico ADMET evaluation of phytochemical derivatives**

To complement the molecular docking studies and further assess the pharmacokinetics properties of the selected derivatives, we evaluated their ADMET properties using in silico predictive models, and the results are presented in Table 3. Most derivatives demonstrated favorable intestinal absorption and high Caco-2 permeability, suggesting good oral bioavailability. For instance, several analogues of Naringenin 5-rhamnoside and Pinostrobin 5-glucoside exhibited absorption percentages above 85%, alongside acceptable skin permeability and volume of distribution (VDss), indicating balanced distribution profiles. Blood-brain barrier (BBB) permeability was generally low across the compounds, which is beneficial in avoiding CNS-related side effects for non-neuroactive targets such as EZH2.

Furthermore, most of the phytochemical derivatives did not inhibit major cytochrome P450 enzymes, including CYP3A4 and CYP2D6, thereby reducing the likelihood of drug-drug interactions. In terms of toxicity, some of the derivatives showed AMES mutagenicity or hepatotoxicity, while some exhibited low probabilities for hERG channel inhibition and drug-induced liver injury (DILI), both critical parameters for cardiac and hepatic safety.

**Table 3:** The adsorption, distribution, metabolism, excretion and toxicology profile of phytochemical derivatives

Phytochemical	Adsorption	1	2	3	4	5	6	7	9	10
Moracin P	Caco2 Permeability (Log cm/s)	− 4.671	− 5.145	− 4.441	− 4.863	− 4.478	− 4.604	− 5.158	− 4.354	− 5.221
	Lipophilicity (LogD)	2.916	2.877	0.788	3.414	2.993	2.944	2.865	3.234	3.121
	Human Intestinal Absorption	0.999	0.999	1	0.963	0.998	0.999	0.996	0.999	0.997
	P-glycoprotein Inhibition	0.002	0.008	0.016	0.972	0.084	0.003	0.003	0.001	0.536
	Lipophilicity (LogP)	3.61764	3.14914	3.2795	3.7982	3.492	2.9261	2.84072	3.21802	3.3297
	*Distribution*									
	Plasma Protein Binding Rate (%)	83.586	87.612	96.249	88.074	75.342	86.764	77.708	95.552	89.682
	Volume of Distribution (L/kg)	2.367	2.882	1.295	2.862	4.255	2.365	2.576	2.576	2.724
	Blood-Brain Barrier Penetration	0.997	0.998	0.885	0.995	0.915	0.519	0.995	1	0.973
	*Metabolism*									
	CYP2C9 Inhibition	0.98	0.469	0.585	0.957	0.739	0.328	0.563	0.662	0.835
	CYP2D6 Inhibition	0.036	0	0.004	0.001	0.013	0.011	0	0	0.003
	CYP3A4 Inhibition	0.139	0.047	0.044	0.943	0.878	0.135	0.007	0.478	0.974
	*Excretion*									
	Clearance Hepatocyte (mL/min/g)	77.662	77.662	77.662	77.662	77.662	77.662	77.662	77.662	77.662
	Clearance Microsome (mL/min/g)	77.662	77.662	77.662	77.662	77.662	77.662	77.662	77.662	77.662
	Half-life in Human (hour)	77.662	77.662	77.662	77.662	77.662	77.662	77.662	77.662	77.662
	*Toxicity*									
	LD50 in Rat (-Log mol/kg)	2.122	2.597	2.285	2.148	2.131	2.378	2.361	2.826	2.756
	AMES Mutagenicity	0.999	1	0.02	0.965	0.998	0.999	1	0.966	0.918
	Drug-Induced Liver Injury	1	1	1	1	1	1	1	0.998	1
	hERG Inhibition	0.002	0.001	0.014	0.354	0.091	0.001	0	0.025	0.72

Naringenin 5-rhamnoside	Absorption	1	2	3	4
	Caco2 Permeability (Log cm/s)	− 5.188	− 5.188	− 5.149	− 5.149
	Lipophilicity (LogD)	1.577	1.577	0.41	0.41
	Human Intestinal Absorption	0.998	0.998	0.998	0.998
	P-glycoprotein Inhibition	0.775	0.775	0.415	0.415
	Lipophilicity (LogP)	3.7925	3.7925	3.1605	3.1605
	*Distribution*				
	Plasma Protein Binding Rate (%)	98.042	98.042	95.78	95.78
	Volume of Distribution (L/kg)	2.344	2.344	2.58	2.58
	Blood-Brain Barrier Penetration	0.751	0.751	0.899	0.899
	*Metabolism*				
	CYP2C9 Inhibition	0.991	0.991	0.997	0.997
	CYP2D6 Inhibition	0.003	0.003	0.001	0.001
	CYP3A4 Inhibition	0.347	0.347	0.033	0.033
	*Excretion*				
	Clearance Hepatocyte (mL/min/g)	50.729	50.729	58.006	58.006
	Clearance Microsome (mL/min/g)	26.365	26.365	47.971	47.971
	Half-life in Human (hour)	176.186	176.186	5.656	5.656
	*Toxicology*				
	LD50 in Rat (-Log mol/kg)	3.449	3.449	3.449	3.449
	AMES Mutagenicity	0.205	0.205	0.205	0.205
	Drug-Induced Liver Injury	0.998	0.998	0.998	0.998
	hERG Inhibition	0.854	0.854	0.854	0.854

Pinostrobin 5-glucoside	Adsorption	1	2	3	5
	Caco2 Permeability (Log cm/s)	− 4.965	− 4.865	− 4.617	− 5.366
	Lipophilicity (LogD)	2.74	1.178	3.756	3.02
	Human Intestinal Absorption	1	1	1	1
	P-glycoprotein Inhibition	0.493	0.454	0.22	0.94
	Lipophilicity (LogP)	3.5948	1.9382	4.42012	3.29864
	*Distribution*				
	Plasma Protein Binding Rate (%)	91.101	80.25	98.81	85.572
	Volume of Distribution (L/kg)	− 0.031	0.788	3.81	0.4
	Blood-Brain Barrier Penetration	0.999	0.715	0.981	0.274
	*Metabolism*				
	CYP2C9 Inhibition	0.721	0.009	0.912	0.603
	CYP2D6 Inhibition	0.127	0	0	0.012
	CYP3A4 Inhibition	1	0.917	0.992	1
	*Excretion*				
	Clearance Hepatocyte (mL/min/g)	77.814	72.307	100.101	69.903
	Clearance Microsome (mL/min/g)	24.512	16.751	64.94	47.812
	Half-life in Human (hour)	15.384	6.313	31.636	12.579
	*Toxicology*				
	LD50 in Rat (− Log mol/kg)	3.104	3.332	0.825	3.660
	AMES Mutagenicity	0.179	0.911	0.905	0.240
	Drug-Induced Liver Injury	0.825	0.905	0.988	0.132
	hERG Inhibition	0.696	0.484	0.132	0.977

Sakuranin	Adsorption	1	2	3	4	5	6	7	8	9	10
	Caco2 Permeability (Log cm/s)	− 6.791	− 6.791	− 6.791	− 6.791	− 6.791	− 4.725	− 4.725	− 4.725	− 4.725	− 4.751
	Lipophilicity (LogD)	1.45	1.45	1.45	1.45	1.45	1.832	1.832	1.832	1.832	1.631
	Human Intestinal Absorption	0.301	0.301	0.301	0.301	0.301	0.998	0.998	0.998	0.998	0.998
	P-glycoprotein Inhibition	0.005	0.005	0.005	0.005	0.005	0.76	0.76	0.76	0.76	0.632
	Lipophilicity (LogP)	0.286	0.286	0.286	0.286	0.286	3.7054	3.7054	3.7054	3.7054	3.7054
	*Distribution*										
	Plasma Protein Binding Rate (%)	74.244	74.244	74.244	74.244	74.244	95.389	95.389	95.389	95.389	97.482
	Volume of Distribution (L/kg)	2.764	2.764	2.764	2.764	2.764	1.532	1.532	1.532	1.532	1.403
	Blood-Brain Barrier Penetration	0.024	0.024	0.024	0.024	0.024	0.928	0.928	0.928	0.928	0.76
	*Metabolism*										
	CYP2C9 Inhibition	0	0	0	0	0	0.305	0.305	0.305	0.305	0.923
	CYP2D6 Inhibition	0	0	0	0	0	0.01	0.01	0.01	0.01	0.002
	CYP3A4 Inhibition	0	0	0	0	0	0.039	0.039	0.039	0.039	0.01
	*Excretion*										
	Clearance Hepatocyte (mL/min/g)	7.031	7.031	7.031	7.031	7.031	49.732	49.732	49.732	49.732	68.527
	Clearance Microsome (mL/min/g)	52.199	52.199	52.199	52.199	52.199	14.147	14.147	14.147	14.147	23.937
	Half-life in Human (hour)	1.516	1.516	1.516	1.516	1.516	7.027	7.027	7.027	7.027	11.054
	*Toxicology*										
	LD50 in Rat (-Log mol/kg)	3.235	3.235	3.235	3.235	3.235	3.204	3.204	3.204	3.204	3.563
	AMES Mutagenicity	0.771	0.771	0.771	0.771	0.771	0.042	0.042	0.042	0.042	0.016
	Drug-Induced Liver Injury	0.9	0.9	0.9	0.9	0.9	0.996	0.996	0.996	0.996	0.993
	hERG Inhibition	0.898	0.898	0.898	0.898	0.898	0.998	0.998	0.998	0.998	0.945

Caco2 Permeability; (perfect: > − 5.15, poor: ≤ − 5.15), Lipophilicity; (perfect: 0.0–3.0 poor: < 0.0 or > 3.0), Human Intestinal Absorption; (perfect: 0.7–1.0, good: 0.3–0.7, poor: 0.0–0.3), P-glycoprotein Inhibition; (perfect: 0.0–0.3, good: 0.3–0.7 poor: 0.7–1.0), Lipophilicity; (perfect: 0.0–3.0, poor: < 0.0 or > 3.0), Plasma Protein Binding Rate; (perfect: ≤ 90, poor: > 90), Volume of Distribution; (perfect: 0.0–20.0, perfect: < 0.0 or > 20.0), Blood-Brain Barrier Penetration; (perfect: 0.0–0.3, good: 0.3–0.7, poor: 0.7–1.0), CYP2C9, CYP2D6, CYP3A4 Inhibitions; (perfect: 0.0–0.3, good: 0.3–0.7, poor: 0.7–1.0), Clearance Hepatocyte and Clearance Microsome; (perfect: ≥ 5, poor: < 5); Half-life in human; (perfect: ≥ 3, Poor: < 3), LD_50_ in Rat; (perfect: ≥ − 2.4, poor: < − 2.4), AMES Mutagenicity; (perfect: 0.0–0.3, good: 0.3–0.7, poor: 0.7–1.0), Drug Induced Liver Injury; (perfect: 0.0–0.3, good: 0.3–0.7, poor: 0.7–1.0), hERG Inhibition; (perfect: 0.0–0.3, good: 0.3–0.7, poor: 0.7–1.0)

### Molecular dynamics simulation

**Fig. 2:**
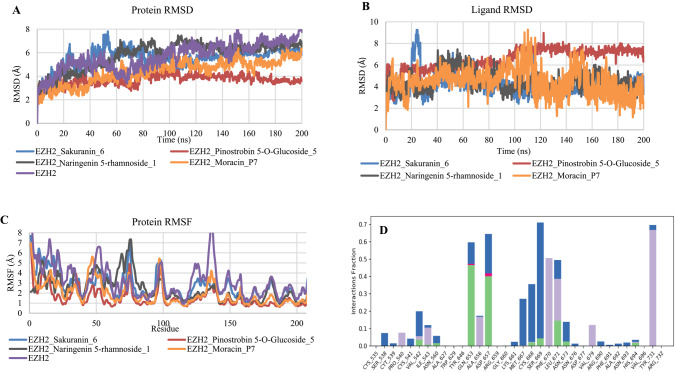

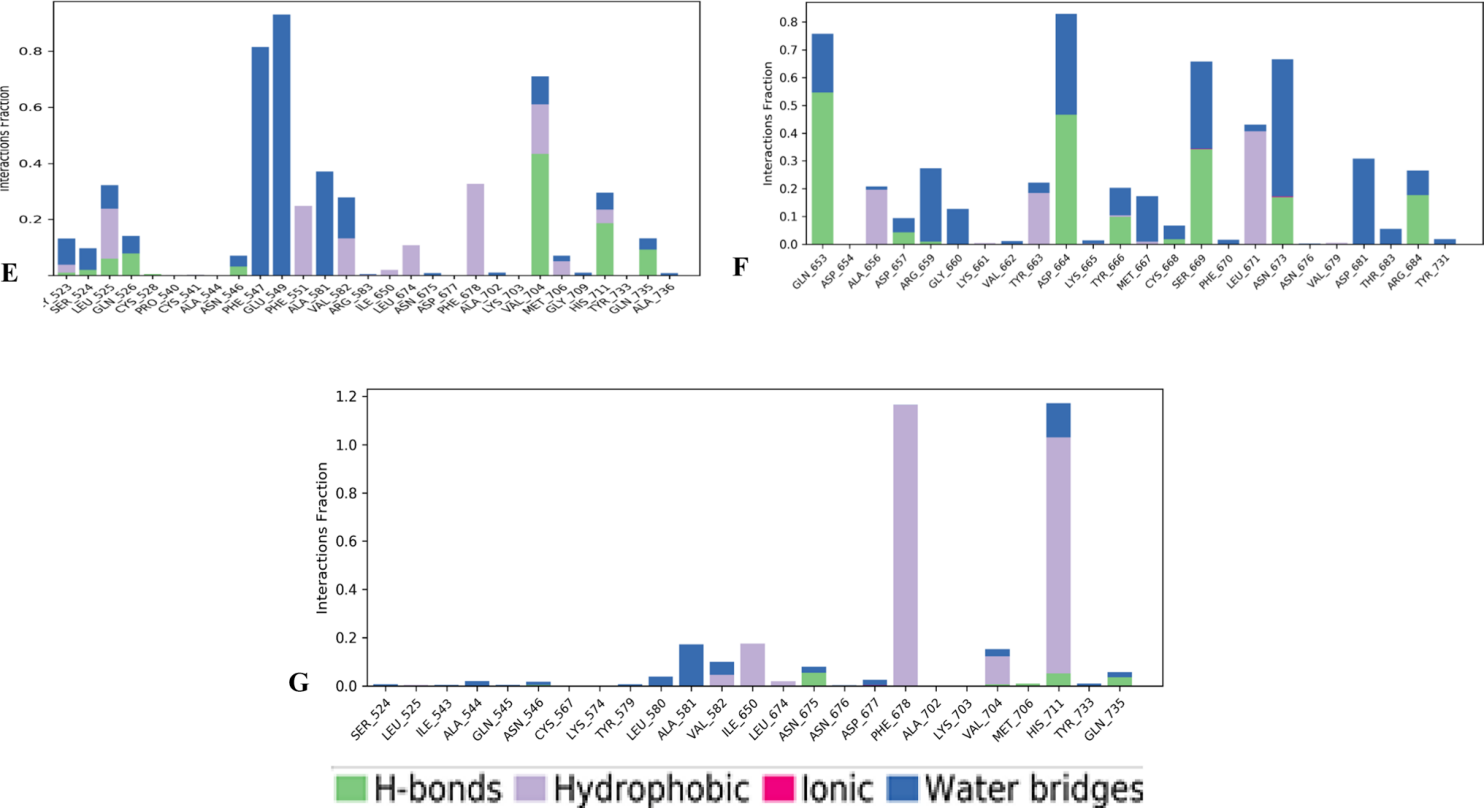
Molecular dynamics simulation analysis of EZH2–ligand complexes. (**A**) RMSD profiles of the EZH2 protein backbone in its unbound form and in complex with Sakuranin_6, Pinostrobin 5-glucoside_5, Naringenin 5-rhamnoside_1, and Moracin_P7, highlighting overall structural stability. (**B**) RMSD trajectories of Sakuranin_6, Pinostrobin 5-glucoside_5, Naringenin 5-rhamnoside_1, and Moracin_P7 during a 200 ns simulation, showing ligand positional stability within the binding pocket. (**C**) RMSF plots comparing residue-level flexibility of EZH2 in its apo form and upon binding to the selected ligands, indicating regions stabilized by ligand interaction. (**D–G**) Protein–ligand contact maps for EZH2 in complex with Sakuranin_6 (**D**), Pinostrobin 5-glucoside_5 (**E**), Naringenin 5-rhamnoside_1 (**F**), and Moracin_P7 (**G**), illustrating key interacting residues and types of molecular contacts contributing to complex stability. Note: Pinostrobin 5-O-Glucoside is synonyms to Pinostrobin 5-glucoside

To evaluate the stability and conformational flexibility of the compounds within the EZH2 active site, the top-performing derivative of each scaffold was subjected to a 200 ns MDS. The results are presented in Fig. 2. As shown in Fig. 2A and 2B, the EZH2–Moracin_P7 and EZH2–Pinostrobin 5-glucoside_5 complexes demonstrated stable protein backbone and ligand RMSD profiles throughout the simulation, indicating consistent binding. In contrast, the EZH2–Sakuranin_6 and EZH2–Naringenin 5-rhamnoside_1 complex exhibited initial stability followed by fluctuations in the mid-simulation phase, before stabilizing again toward the end of the 200 ns trajectory.

Root Mean Square Fluctuation (RMSF) analysis revealed that several residues exhibited reduced flexibility in the ligand-bound state compared to the apo form, particularly in regions surrounding the binding pocket (Fig. 2C). Furthermore, protein–ligand contact analysis (Fig. 2D-G) identified key residues contributing to complex stability and the nature of interactions involved, including hydrogen bonds and hydrophobic contacts.

## Discussion

In this study, we built upon our previous research, in which we identified five phytochemicals: Moracin P, Naringenin 5-rhamnoside, Phytocassane A, Pinostrobin 5-glucoside, and Sakuranin as potential inhibitors of EZH2. Notably, these phytochemicals exhibited higher predicted binding affinities for EZH2 than known inhibitors such as GSK126 and Benzafibrate. To further these findings, we utilized the CHEESE webserver, which employs the SMILES representations of these phytochemicals to generate structurally related derivatives. These derivatives were subsequently evaluated for their predicted binding affinities, drug-like properties, pharmacokinetic profiling, and toxicity prediction, to identify compounds with improved inhibitory potential against EZH2, and thus therapeutic relevance in PC.

As evident in Table 1, the derivatives of Moracin P exhibited exceptionally high predicted bioavailability, with scores ranging from 0.984 to 1, while also maintaining favorable physicochemical parameters, including low topological surface area (TPSA < 120 Å^2^), moderate molecular weights below 500, good lipophilicity values (ClogP < 5) and molar refractivity between the acceptable range (40–130). Interestingly, all derivatives of moracin P retained physicochemical properties like those of the parent compound moracin P and exhibited TPSA below 120 Å^2^, acceptable molar refractivity and no hydrogen bond donors except Z1123898030 (moracin P_3) with one hydrogen bond donor, while the parent compound moracin P had three hydrogen bond donors.

The derivatives of Naringenin 5-rhamnoside, including ZINC001226496196-a (Naringenin 5-rhamnoside_1) and ZINC001226496195-a (Naringenin 5-rhamnoside_2), exhibited an average predicted bioavailability of 0.579. In contrast, the remaining derivatives showed good bioavailability of 0.706–0.858, with hydrogen bond donors between one to three compared to the parent compound with five hydrogen donors. All derivatives of Naringenin 5-rhamnoside have TPSA below 120 Å^2^, and an acceptable molar refractivity range.

Moreover, all derivatives of Phytocassane A demonstrated poor bioavailability (< 0.300) except ZINC000253497334 (Phytocassane A_5), ZINC000253497332 (Phytocassane A_6), ZINC000253497333 (Phytocassane A_7), and ZINC000004612836 (Phytocassane A_8) with an average bioavailability of 0.517. All derivatives have two hydrogen bond donors each, TPSA below 120 Å^2^ and acceptable molar refractivity compared to the parent compound Phytocassane A.

On the other hand, derivatives of Pinostrobin 5-glucoside exhibited higher predicted bioavailability (0.987-1), TPSA below 120 Å^2^, an acceptable molar refractivity range and one or two hydrogen bond donors, except Z2160012302 (Pinostrobin 5-glucoside 3) with no hydrogen donor. This suggests an improved pharmacokinetic potential of the parent compound.

Interestingly, ZINC000005742781 (Sakuranin_1), ZINC000005742784 (Sakuranin_2), ZINC000005742783 (Sakuranin_4), ZINC000005742785 (Sakuranin_4), and ZINC000016951757 (Sakuranin_5) derivatives of Sakuranin exhibit average predicted bioavailability (0.304), five hydrogen donors each and TPSA above 120 Å^2^, while the other have good, predicted bioavailability between 0.963 and 0.970, one hydrogen donor each and TPSA below 120 Å^2^. Overall, all derivatives have an acceptable molar refractivity range within 40–130.

In addition to the promising physicochemical and bioavailability profiles observed in the derivatives, we also leveraged molecular docking to predict the binding affinity of the derivatives. While the derivatives exhibited varying degrees of affinity for EZH2, some of the compounds exhibited higher predicted binding affinity than the original compound, as evident from their docking score presented in Table 2. The derivatives of moracin P demonstrated consistently stronger predicted binding affinities with docking scores ranging from − 6.8 to − 7.7 Kcal/mol, notably outperforming the original compound, which had a docking score of − 7.6 Kcal/mol, with Moracin P_2 and Moracin P_7 exhibiting the highest affinity. Interestingly, Moracin P_1, Moracin P_3, Moracin P_4, Moracin P_5, Moracin P_8, Moracin P_9 and Moracin P_10 had a docking score lower than that of the parent compound (− 7.6 Kcal/mol).

Similarly, Naringenin 5-rhamnoside derivatives displayed binding affinities between − 7.8 and − 8.2 Kcal/mol; however, only the first four derivatives showed improvements in binding affinities, all with docking scores of − 8.2 Kcal/mol. Phytocassane A recorded the least binding score among the five leads with a docking score of − 6.8 Kcal/mol, but all of its derivatives demonstrated high binding affinity of − 7.2 Kcal/mol, particularly the Phytocassane A_1–5 derivatives, with lower predicted binding affinities of − 7.1 Kcal/mol for Phytocassane A_9–10, suggesting the structures of the derivatives have diverged in scaffolds that are crucial to EZH2 interaction.

Contrastingly, only Pinostrobin 5-glucoside_5 exhibited better predicted binding affinities (− 8.1 S) than the parent compound Pinostrobin 5-glucoside (− 7.7 Kcal/mol), with other derivatives having docking scores ranging from − 6.4 to − 7.6 Kcal/mol. Finally, all derivatives of Sakuranin consistently exhibited low predicted binding affinities (− 7.2 Kcal/mol) below parent template (− 7.6 Kcal/mol) docking scores. All derivative ranges between − 7.2 to − 7.7 Kcal/mol.

The binding interactions of selected phytochemical derivatives with the active site of EZH2 were visualized and analysed (Fig. 1). Distinct scaffolds demonstrated unique interaction profiles with key amino acid residues, revealing potential binding stability and specificity. Moracin_P2 formed a hydrogen bond with ARG583 and exhibited hydrophobic interactions with multiple residues including Gly523, Ser524, Leu525, Gln526, Asn546, Phe547, Glu549, Phe551, Val582, Val704, Met706, and His711 (Fig. 1A). Similarly, Moracin_P7 engaged in hydrogen bonding with Asn675 and His711, and hydrophobic interactions with Ser524, Val582, Ile650, Phe678, Val704, Met705, Met706, and GLN735 (Fig. 1B). Naringenin 5-rhamnoside_1 interacted with EZH2 through hydrogen bonds with Asp664 and Asn673, in addition to hydrophobic contacts involving Ala656, Arg659, Gly660, Val662, Tyr666, Met667, Leu671, Phe672, Ser669, and Val679 (Fig. 1C). Phytocassane A_1 formed hydrogen bonds with Arg583, Asn675, and Gln735, along with hydrophobic interactions with residues such as Asn546, Phe547, Ala581, Val582, Phe678, Val704, Met706, and His711 (Fig. 1D). Meanwhile, Phytocassane A_5 formed hydrogen bonds with Gln653, Asn673, and His694, and hydrophobically interacted with Ala656, Asp657, Leu671, Val679, Tyr731, and Arg732 (Fig. 1E). Pinostrobin-5-glucoside_5 primarily formed hydrophobic interactions with EZH2 residues, including Gly523, Ser524, Leu525, Gln526, Pro540, Cys541, Ala544, Asn546, Phe547, Glu549, Ala581, Arg583, Val704, Met706, His711, and Gln735 (Fig. 1F). Lastly, Sakuranin_6 established a hydrogen bond with Asp657 and hydrophobic interactions with Tyr646, Ala656, Ser669, Phe670, Leu671, Arg690, and Tyr731 (Fig. 1G).

Following the interaction profiling, we sought to further understand the therapeutic viability of the derivatives by evaluating their pharmacokinetic properties. Interestingly, all the evaluated derivatives showed high human intestinal absorption potential, except for the first five derivatives of Sakuranin. Additionally, these compounds also exhibited high predicted Caco2 permeability potential, suggesting they can all be administered via the oral route, except the first five derivatives of Sakuranin. Notably, Sakuranin_1 to Sakuranin_5 had poor predicted human intestinal absorption, which is also in tandem with the results of the Caco2 permeability potential.

Following the interaction profiling, we further assessed the therapeutic viability of the derivatives by evaluating their pharmacokinetic properties. Interestingly, all the evaluated compounds demonstrated high predicted human intestinal absorption and Caco-2 cell permeability, suggesting their potential for oral administration. However, a notable exception was observed with Sakuranin derivatives 1 through 5, which exhibited poor human intestinal absorption (HIA) and low Caco-2 permeability predictions. In addition to the intestinal absorption potential of compounds, another critical factor that influences their effectiveness is their interaction with the P-glycoprotein (P-gp). Notably, the P-gp is an ATP-dependent efflux transporter involved in modulating drug absorption, distribution, and excretion. It plays.

The derivatives also displayed notable variability in their predicted ability to inhibit P-glycoprotein (P-gp), an ATP-dependent efflux transporter involved in modulating drug absorption, distribution, and excretion. P-gp plays a crucial role in limiting drug accumulation in cells, particularly in tissues such as the intestine, liver, kidneys, and blood–brain barrier, and its inhibition can have dual implications. Among the moracin P derivative, only moracin P_4 was identified as a potential P-gp inhibitor, suggesting a potential for enhanced intracellular drug retention and reduced efflux-mediated resistance, which could be advantageous in cancer therapeutics. Likewise, Naringenin 5-rhamnoside_1 and Naringenin 5-rhamnoside_2 from the Naringenin-5-rhamnoside group, as well as Pinostrobin 5-glucoside_5 from the Pinostrobin 5-glucoside series, were also predicted to inhibit P-gp. Notably, Sakuranin derivatives 6 through 9 exhibited similar inhibitory profiles. While P-gp inhibition may improve oral bioavailability and therapeutic efficacy by increasing drug concentration at the target site, it could also pose risks such as increased toxicity, adverse drug–drug interactions, and reduced clearance of xenobiotics and endogenous compounds. Consequently, the administration of these compounds may require careful consideration of potential interactions and adjustment of dosages to minimize the risk of adverse effects (Karthika and Sureshkumar 2021).

With regards to the distribution of the compounds after administration, we assessed this by predicting parameters including the plasma protein binding rate, volume of distribution, and blood-brain barrier penetration.

The plasma protein binding rate of the derivatives is an essential factor in determining the pharmacokinetics and efficacy of a drug. The derivatives of Moracin P, Naringenin 5-rhamnoside, Pinostrobin 5-glucoside, and Sakuranin all exhibit high protein binding percentages, ranging from 74% to 98%. While this indicates that the drugs may potentially remain in circulation for longer periods due to slow elimination, it may also reduce the free drug concentration that can interact with the target, EZH2. For clinical development, binding rates above 90% are often considered suboptimal, as they may result in a high proportion of the drug being bound to plasma proteins and therefore unavailable for therapeutic action. However, in certain cases, prolonged circulation time is beneficial, especially if the drug needs to accumulate at the target site over time. Among our derivatives, Moracin P and Sakuranin derivatives exhibit a more favorable range (75–90%), which suggests that they might offer an ideal balance between sufficient drug half-life and availability for biological activity. Conversely, Pinostrobin 5-glucoside and Naringenin 5-rhamnoside derivatives exhibited higher predicted binding rates (> 95%).

We also evaluated the potential of the enzymes to inhibit important phase I metabolism enzymes, which mediate the metabolism of xenobiotics. Inhibition of cytochrome P450 enzymes, particularly CYP2C9, CYP2D6, and CYP3A4, represents a critical pharmacokinetic liability, as it can lead to metabolic interference, adverse drug-drug interactions, and altered therapeutic outcomes (Deodhar et al. 2020). Therefore, we evaluated the predicted inhibitory potentials of the most promising derivatives of each phytochemical lead compound to assess their metabolic compatibility.

Among the Moracin P derivatives, CYP2D6 inhibition remained negligible across all candidates, with values ranging from 0.000 to 0.036, suggesting minimal risk of interfering with substrates metabolized via this isoform. However, most derivatives demonstrated poor inhibition profiles against CYP2C9 and CYP3A4, with inhibition probabilities often exceeding 0.7, indicative of potential metabolic risks. Notably, derivatives 6 and 7 displayed comparatively favorable inhibition values against CYP2C9 (0.328 and 0.563, respectively) and CYP3A4 (0.135 and 0.007), highlighting them as the most metabolically viable among the Moracin series. For Naringenin 5-rhamnoside derivatives, although CYP2D6 and CYP3A4 inhibition values were within acceptable ranges (0.001–0.003 and 0.033–0.347, respectively), all four derivatives showed poor inhibition profiles for CYP2C9, with values exceeding 0.99. This suggests a high likelihood of interfering with CYP2C9-mediated metabolism and limits their administration with CYP2C9 inhibitors. Pinostrobin 5-glucoside derivatives presented a less favorable profile, with strong inhibition of CYP3A4 observed in all candidates, with values ranging from 0.917 to 1.0. While some derivatives exhibited minimal CYP2D6 inhibition and acceptable CYP2C9 interaction, the consistent inhibition of CYP3A4, which is a key enzyme responsible for metabolizing most drugs, raises significant concerns about their potential to cause drug-drug interactions upon administration. In contrast, Sakuranin derivatives showed a markedly superior CYP inhibition profile. Derivatives 1 through 9 demonstrated perfect to good inhibition ranges across all three CYP isoforms. Specifically, CYP2C9 and CYP2D6 inhibition values were uniformly low (0.0–0.305 and 0.0–0.01, respectively), and CYP3A4 inhibition remained near-zero (0.0–0.039). Only derivative 10 exhibited poor CYP2C9 inhibition (0.923).

In furtherance of the evaluation of the potential of the derivatives to serve as EZH2 inhibitors, we assessed their toxicity properties. Toxicological assessment is critical in early drug discovery to preclude candidates with potential safety liabilities (Chukwuemeka et al. 2021). Accordingly, in silico predictions of rat oral LD_50_, AMES mutagenicity, drug-induced liver injury (DILI), and hERG inhibition were employed to evaluate the safety profiles of the lead derivatives.

Among the Moracin P derivatives, six compounds exhibited rat LD_50_ values below the optimal threshold of -2.4 log(mol/kg), suggesting potential acute toxicity risks. However, Moracin P_2 (LD_50_ = 2.597), Moracin P_9 (LD_50_ = 2.826), and Moracin P_10 (LD_50_ = 2.756) showed relatively improved profiles. AMES mutagenicity predictions revealed a concerning trend, with most derivatives exhibiting high mutagenic potential (0.918–1.000), except Moracin P_3 (0.02), which fell within the ideal range. In addition to the poor AMES outcomes, all derivatives showed high DILI risk (≥ 0.998), and only a few, notably Moracin P_1–3 and Moracin P_6–7, demonstrated minimal hERG inhibition (< 0.03), suggesting a reduced potential of cardiotoxicity for those candidates (Lee et al. 2019). Conversely, Moracin P_10 showed a high hERG inhibition score (0.72), flagging potential cardiotoxic concerns.

For the Naringenin 5-rhamnoside derivatives, all had acceptable LD_50_ values (3.449). The AMES mutagenicity prediction suggests they do not possess mutagenicity potential, however, the DILI and hERG inhibition showed high potential to induce liver injury and cardiotoxicity. Pinostrobin 5-glucoside derivatives presented a mixed profile. All the derivatives had predicted LD_50_ scores well above the acceptable threshold, while only derivatives 1 and 5 were predicted to be non-mutagenic. Additionally, only compound 5 showed no DILI risk, while others, except derivative 3, showed no potential cardiotoxic concern.

Sakuranin derivatives displayed the most favorable toxicity profile among the candidates. All ten derivatives had high LD_50_ values (3.204–3.563), indicating low acute toxicity. Although derivatives 1–5 had high AMES mutagenicity values (0.771), derivatives 6–10 showed improved mutagenic profiles (0.016–0.042), within acceptable thresholds (Zeiger 2019). The predicted DILI potential was poor for all the compounds, and the potential to inhibit the hERG.

After evaluating all the protein–ligand complexes, four were selected for MDS (EZH2_Sakuranin_6, EZH2_Pinostrobin 5-glucoside_5, EZH2_Naringenin 5-rhamnoside_1, and EZH2_Moracin_P7) based on their predicted binding affinities. In instances where multiple derivatives from the same parent compound exhibited identical docking scores, a single representative compound was chosen for further analysis. The protein complexes with Moracin_P7 and Pinostrobin 5-glucoside_5 displayed notably lower RMSD fluctuations compared to the apo form, suggesting that these ligands stabilized the protein structure throughout the simulation (Yu et al. 2024). In contrast, the EZH2_Sakuranin_6 complex showed a behaviour like that of the apo protein during the first 40 ns. Between 40 ns and 100 ns, higher RMSD values were recorded, indicating a period of transient instability. However, after 100 ns, the protein stabilized, with RMSD fluctuations lower than those of the apo form. Similarly, for EZH2_Naringenin 5-rhamnoside_1, the protein’s dynamics were comparable to the apo protein during the first 40 ns. Between 40 ns and 140 ns, more pronounced fluctuations were observed before the complex stabilized, with lower variations than those of the unbound protein. This dynamic trend aligns with previous findings where protein–ligand complexes often display an initial period of instability, typically due to ligand accommodation or induced fit, before reaching equilibrium and showing sustained structural stability over longer simulation times (Alshahrani 2025).

The analysis of the ligands’ RMSD profiles within their complexes with EZH2 provided insights into their stability throughout the MDS. As shown in Fig. 2, the ligands exhibited distinct behaviours in terms of fluctuations and stabilization within the EZH2 active site. For the EZH2_Sakuranin_6 complex, the ligand’s RMSD plot revealed significant fluctuations during the first 120 ns, with a notable peak between 20 and 30 ns where the RMSD reached approximately 9 Å, indicating substantial conformational rearrangements of the ligand. After 125 ns, the system stabilized with minimal RMSD fluctuations, reflecting equilibrium conformational behaviour consistent with observations in similar protein–ligand systems, where initial ligand repositioning transitions into stabilization (Wu et al. 2024).

In the case of EZH2_Pinostrobin 5-O-Glucoside_5, the ligand RMSD initially ranged between 3 and 6 Å during the first 10 ns, before stabilizing around 6 Å up to 95 ns. Beyond this point, the RMSD slightly increased to about 7 Å, after which the complex remained relatively stable, showing only minor residual fluctuations during the rest of the trajectory. The EZH2_Naringenin 5-rhamnoside_1 complex displayed greater flexibility during the first 100 ns, with RMSD values ranging from 3 to 7.5 Å, reflecting successive conformational adjustments of the ligand within the binding site. After 100 ns, the complex reached a more stable state, with the RMSD exhibiting minimal fluctuations. Finally, for the EZH2_Moracin_P7 complex, the ligand remained largely stable throughout the simulation, with only slight fluctuations observed between 100 and 120 ns, suggesting that the ligand maintained its conformation well within the EZH2 active site (Ashraf et al. 2022).

Among the various properties evaluated through MDS to assess protein stability, the RMSF analysis of Cα atoms was performed. This analysis tracks the mobility of each residue throughout the entire simulation, allowing a detailed comparison between the unbound (apo) protein and the protein–ligand complexes, as illustrated in Fig. 2. In general, most residues in the ligand-bound complexes exhibited lower fluctuations compared to the apo form, indicating enhanced structural stability upon ligand binding. However, some exceptions were noted. In the apo form, several residues displayed significant fluctuations ranging from 7.3 to 9.7 Å. These include Gly523, Ser524, Ser537, Tyr663, Asp664, and Lys665, suggesting that these regions are particularly flexible in the absence of a ligand. For the EZH2_Moracin_P7 complex, Gly523 showed the highest fluctuation among the residues, with a value of 6.7 Å. Nonetheless, all other residues exhibited more stable behaviour compared to the apo form, suggesting an overall stabilizing effect of Moracin P7. In the EZH2_Naringenin 5-rhamnoside_1 complex, a few residues showed higher fluctuations than in the apo form. These include Val604, Asp597, Ala595, Ala596, Leu591, Ser605, Thr592, Gly594, Arg532, Cys590, Pro531, and Cys606, with fluctuation values ranging between 4.77 Å and 7.31 Å. These localized increases in flexibility may reflect conformational adjustments upon ligand binding, although the rest of the protein remained stable. For the EZH2_Sakuranin_6 complex, Gly523 again exhibited the highest fluctuation, reaching 7.68 Å. Additionally, Asp597, Asp529, Ala596, and His530 also displayed notable fluctuations, ranging from 5.89 to 7.11 Å, indicating localized flexibility. Finally, in the EZH2_Pinostrobin 5-glucoside_5 complex, only Gly523 showed a significant fluctuation of 6.9 Å, while all other residues remained stable, with fluctuations not exceeding 4 Å.

To gain a better understanding of the interaction mechanism between the studied ligands and the EZH2 target protein, a protein–ligand contact analysis was carried out. As illustrated in Fig. 2, this analysis made it possible to identify the key amino acid residues involved in ligand binding, as well as the types of non-covalent interactions that contribute to the stability of the complexes. The main interactions observed included hydrogen bonds, hydrophobic contacts, water bridges, and in some cases, ionic interactions. Altogether, these forces significantly contribute to the affinity and specificity of the ligands toward the active site of EZH2.

In the EZH2_Sakuranin_6 complex, the residues Gln653, Asp657, Ser669, and Tyr731 exhibited contact fractions greater than 0.6, indicating a strong involvement in ligand anchoring. These residues formed a network of interactions consisting of hydrogen bonds, hydrophobic contacts, and water bridges, contributing to the stable and specific binding of the ligand. In the EZH2_Pinostrobin 5-glucoside_5 complex, the residues Phe547, and Glu549 also showed high contact fractions, exceeding 0.6, with interactions mainly stabilized through water bridges. Additionally, residue Val704 participated in multiple types of interactions, including hydrogen bonds, hydrophobic contacts, and water-mediated bridges, further enhancing the overall stability of the complex. For the EZH2_Naringenin 5-rhamnoside_1 complex, residues Gln653, Asp664, Ser669, and Asn673 were the most involved in ligand interaction. Their high contact fractions, along with the presence of hydrogen bonds and water bridges, suggest strong electrostatic complementarity between the molecule and the protein surface. Finally, in the EZH2_Moracin_P7 complex, residue Phe678 was primarily involved in hydrophobic interactions, while His711 simultaneously formed hydrophobic contacts, hydrogen bonds, and water bridges. This behaviour suggests a key role for His711 in stabilizing and orienting the ligand within the active site of EZH2.

## Conclusion

This study highlights the phytochemical derivatives generated from CHEESE webserver for epigenetic modulators targeting EZH2, a histone methyltransferase frequently overexpressed in various malignancies. Using a structure-based drug discovery strategy that included molecular docking, pharmacokinetics and toxicity profiling, and 200-nanosecond MDS, we identified and characterized several derivatives of phytochemicals, including Moracin P, Naringenin 5-rhamnoside, Sakuranin, Pinostrobin 5-glucoside, and Phytocassane A. These phytochemical derivatives demonstrated strong and specific interactions with EZH2 at key amino acids in the active site, displayed favorable drug-likeness, ADMET properties, and maintained stable binding profiles throughout the simulation, indicating they can be explored as feasible candidate in EZH2 inhibitory study.

Additionally, because cancer requires a broader understanding of the molecular interactions and systemic effects of these phytochemicals and their derivatives towards other potential protein targets, we recommend the application of network pharmacology approaches, to provide valuable insights into the phytochemical derivatives potential to modulate multiple targets and biological pathways involved in cancer progression. In conclusion, this study provides a basis to consider CHEESE webserver for rapid ligand-based screening to support computational foundation for the development of phytochemical-based inhibitors of EZH2 and other cancer target of relevance. Further research incorporating experimental validation is essential to fully uncover their therapeutic potential and guide their progression toward clinical applicability in cancer treatment.

## Supplementary Information

Below is the link to the electronic supplementary material.

Supplementary Material 1

Supplementary Material 2

Supplementary Material 3

Supplementary Material 4

Supplementary Material 5

## Data Availability

All data generated in this study are included in the manuscript and supplementary files.
